# Supplementary material for: Venomix: a simple bioinformatic pipeline for identifying and characterizing toxin gene candidates from transcriptomic data
Source: PeerJ. 2018 Jul 31;6:e5361. doi: 10.7717/peerj.5361 (PMC6074769; doi:10.7717/peerj.5361)
Supplement: Supplemental Information 6 [file peerj-06-5361-s006.gz › FinalOutput_1E-6/Snaclec_echicetin_subunit_beta_6/finaltree.pdf]

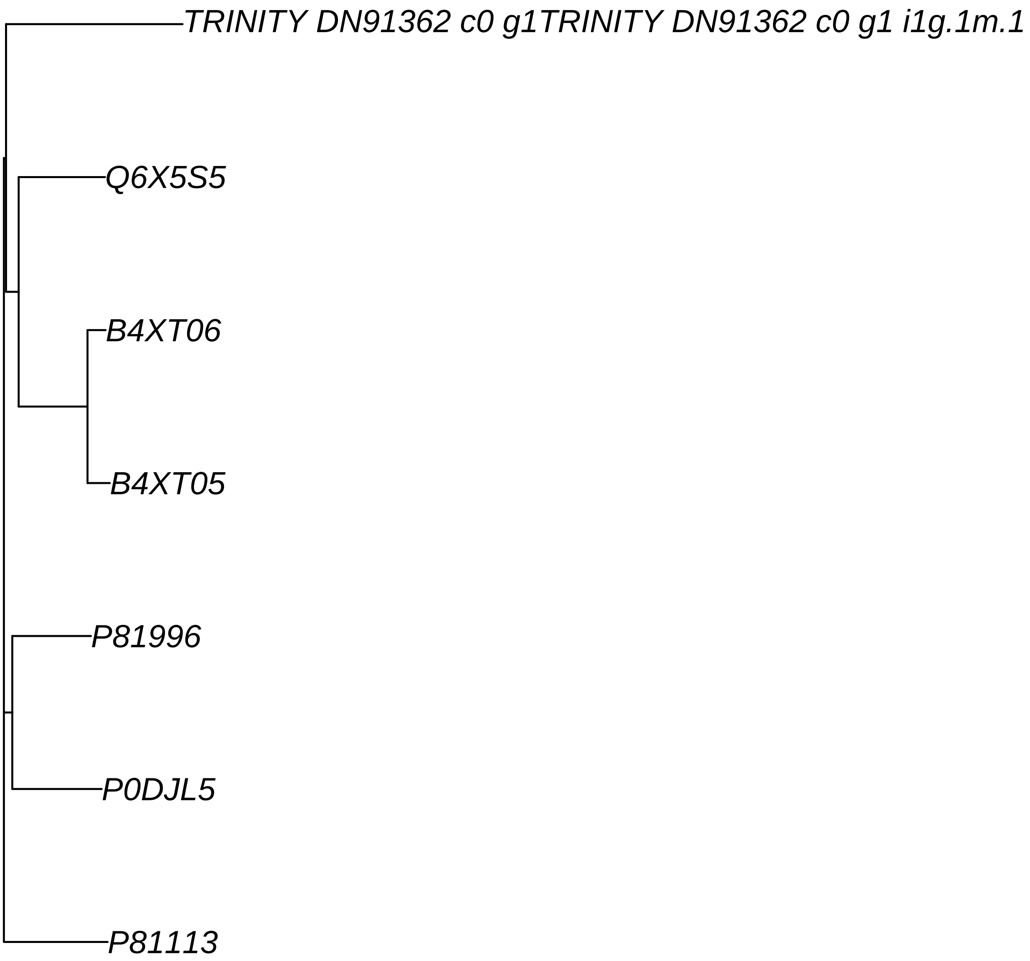

TRINITY DN91362 c0 g1TRINITY DN91362 c0 g1 i1g.1m.1
Q6X5S5
B4XT06
B4XT05
P81996
P0DJL5
P81113
